# Promoting behavioural change by educating anaesthetists about the environmental impact of inhalational anaesthetic agents: A systematic review

**DOI:** 10.1177/0310057X241263113

**Published:** 2024-08-30

**Authors:** Brieana C Nolan, Michael J Hoskins, Bríd Phillips, Kiah L Evans

**Affiliations:** 1 Discipline of Health Professions Education, The University of Western Australia, Crawley, Australia; 2 Department of General Medicine, Sir Charles Gairdner Hospital, Nedlands, Australia; 3 Centre for Arts, The University of Western Australia, Crawley, Australia

**Keywords:** Anaesthetists, global warming, climate change, anaesthetics, inhalation

## Abstract

Of the total carbon footprint of Australia, 7% is attributed to healthcare. In the UK, inhalational agents make up 5% of the healthcare carbon footprint. This systematic review aims to determine which methods of education about the environmental impact of inhalational anaesthetic agents can be utilised to promote behaviour change, reducing the anaesthetic-related carbon footprint. This systematic review sourced records from CINAHL, EMBASE, ERIC, JBI and MEDLINE from 1970 to March 2022. The search identified 589 records, 13 of which met eligibility criteria after the screening process, in which 10 of these records were conference abstracts. Education curricula focused on inhalational agent choice (69%), lowering the fresh gas flow during maintenance anaesthesia (69%), encouraging alternatives such as total intravenous anaesthesia (23%) and/or switching off the gas on transfer (8%). The most common teaching techniques utilised in education curricula were didactic lectures (85%), visual prompts (54%), emails (46%), and conversation forums (31%). All but one study reported a positive relationship between teaching sessions and behavioural change resulting in lower inhalational anaesthetic use by participants and their organisations, reducing healthcare-associated emissions. This systematic review has demonstrated that single education sessions as well as multi-focused, multimodal education curricula on the topic of greener anaesthesia can be beneficial in promoting behavioural change.

## Introduction

Climate change is a key environmental issue faced by our society.^1^ Healthcare workers should have great interest in the impending ecological crisis as they will inevitably be caring for patients and communities whose health will be impacted by the confounding effects of climate change.^2^ Of the total carbon footprint of Australia, 7% is attributed to healthcare, with hospitals and pharmaceuticals being the major contributors.^3^ The specific carbon footprint of anaesthetic agents in Australia is unknown. In the UK, 5% of the total healthcare carbon footprint is attributed to inhalational agents.^4^ This study combined the carbon footprint of volatile anaesthetic agents, nitrous oxide and metered dose inhalers. Of note, this included hospital-wide use of nitrous oxide including obstetrics, dental and emergency departments that are likely to be large contributors to the carbon footprint.

All commonly used modern inhalational anaesthetic agents are recognised greenhouse gases exhibiting negative environmental effects.^5^ Ozone depleting effects, global warming potential (GWP) and atmospheric lifetime are used to explain and compare the environmental impact of inhalational anaesthetic agents (Table 1).^6–8^ GWP refers to a gas’s ability to trap heat in the atmosphere over time relative to carbon dioxide (CO_2_). Atmospheric lifetime refers to the time the gas remains in the atmosphere before equilibrium is reached.

**Table 1.** Ozone depleting effects,^6^ 100-year GWP^7,8^ and atmospheric lifetime^7,8^ of common inhalational anaesthetic agents.

Agent	Ozone depleting effects	100-Year GWP	Atmospheric lifetime
Desflurane	None	2540	14
Isoflurane	None	510	3.2
Nitrous oxide	Yes	265	121
Sevoflurane	Yes	130	1.1

GWP: global warming potential.

While optimising the environmental impact of general anaesthesia, patient quality of care should not be compromised. The inhalational route is the most common route of general anaesthesia;^9–15^ however, alternative routes, such as total intravenous anaesthesia, are safe and effective.^9^ Surveys have showed that there is a lack of knowledge among anaesthetists regarding the negative environmental impacts of inhalational anaesthetic agents, and it is not always a consideration among an anaesthetists’ practice.^10,15–17^ Anaesthesiology colleges around the world have developed guidelines to encourage sustainable anaesthesia, while maintaining patient safety.^18–22^ Reductions in the carbon footprint of anaesthetic care can be made through: shifting away from the use of desflurane and nitrous oxide, and ideally away from the use of all inhalational anaesthetic agents; reducing inhalational anaesthetic agent waste; and the use of closed-circuit low-flow inhalational anaesthesia.^22^

Anaesthetists are in a prime position to be able to modify their practices to minimise their professional carbon footprint. Lack of education and support from the hospital system are key barriers to environmentally sustainable anaesthesia practice.^23^ Unfortunately, many anaesthetic trainees do not receive formal training on environmental sustainability in anaesthesia.^23,24^ Education curricula providing knowledge and skills to anaesthetists about the negative impacts of inhalational agents on the environment are essential for change in behaviour to occur.

The healthcare system commonly allows and encourages doctors to meet their continuing professional development standards through attending education sessions run by hospital staff. Within the hospital system, common teaching techniques include formal lectures, informal or ‘on the run’ sessions, peer-to-peer discussions, online modules, workshops, simulations, and independent reading.^25^ Each of these education techniques aim to cover a varying degree of objectives, each aiming to reach a specific level of the cognitive, psychomotor, and affective process.^25^ Multifaceted education curricula with continuous education targeting barriers to change are more likely to be effective than single interventions.^26^ To optimise learning, it is important that all educators are conscious of the learning styles their students may identify with. The four core learning styles are visual, auditory, reading, and kinaesthetic.^27^ Most learners are versatile and can learn through various methods, but usually have a preferred style. A multimodal approach is the gold standard for enhancing learning across a broad range of learners.^25^ A survey of anaesthetic trainees showed that common formats of exposure to greener anaesthesia were independent reading, peer-to-peer discussions and conference lectures.^23^ Formal curriculum training, conference lectures, peer-to-peer discussions and an online e-module were perceived to be the most valuable methods of education on the topic.^23^

This systematic review aims to determine which methods of education about the environmental impact of inhalational anaesthetic agents can be utilised to promote behaviour change, reducing the carbon footprint of anaesthetic care.

## Review methodology

The goal was to synthesise all available international evidence to inform clinical practice.^28^ A systematic literature review was conducted according to, and the manuscript thus adheres to, the preferred reporting items for systematic reviews and meta-analyses (PRISMA) guidelines.^29^ PROSPERO (international prospective register of systematic reviews) is a database that accepts prospective registrations for review projects, which allow authors access to research currently being undertaken or soon planned to be undertaken.^30^ A search of the PROSPERO database prior to commencement confirmed no similar review was underway. Guiding questions of the systematic review were:

- What are the focuses of education curricula to optimise the environmental impact of inhalational anaesthetic agents?
- What are the education techniques utilised to optimise the environmental impact of inhalational anaesthetic agents?
- What are the ways of measuring the effect of an education curriculum on the environmental impact of inhalational anaesthetic agents?
- Does educating anaesthetists about the environmental impact of inhalational anaesthetic agents result in a change in their behaviour and/or overall anaesthesia carbon footprint?

### Eligibility criteria

The population, intervention and outcome (PIO) and population, exposure, outcome (PEO) frameworks suggest that a good clinical question typically identifies a problem or population, intervention or exposure, and outcome.^31,32^ The eligibility criteria for the systematic review were created based on the PEO framework, with the population studied being practising anaesthesia providers undergoing education detailing the environmental impact of inhaled anaesthetics, with the outcome of interest being behavioural change following intervention.^33^ Inclusion criteria were records: published between 1970 and March 2022; available in English; describing any intentional, pre-arranged education curricula undertaken by anaesthesia providers in a hospital setting about inhalational anaesthetic agents and their environmental impact; involved an evaluation of this education curricula; and included any research methodology (qualitative, quantitative, or mixed) or publication type (including journal articles, conference abstracts, or theses/dissertations). Exclusion criteria were records that do not describe the education curriculum in detail (e.g. insufficient information about content and/or teaching methods); or did not present original research.

### Information sources and search strategy

A systematic search of CINAHL, EMBASE, ERIC, JBI, and MEDLINE databases was performed in March 2022 using a combination of EBSCOhost, Ovid and ProQuest search platforms. The search included literature published between 1970 and March 2022. The year 1970 was selected as the cut-off, as this is the decade when research about the negative impact of anaesthesia on the environment commenced.^34–36^ The key search terms used for the search strategy are seen in Supplementary Table 1. The search was completed and collated by the first author using Microsoft Excel (Microsoft Corporation, Redmond, WA, USA). Duplicates were removed prior to title and abstract review.

### Selection process

The titles and abstracts of identified studies were reviewed independently by both the first and second authors to assess whether they met the eligibility criteria to be included in the full-text review. If abstracts were unable to be imported alongside the title from the database search, then they proceeded to full-text review automatically. At the full-text review stage, both of these authors reviewed manuscripts’ text in full to assess for eligibility to progress to be included in the systematic review. The reason for exclusion at the full text review stage was noted (Supplementary Table 2). Consensus was reached at each stage of the process, with the third author available to solve disputes regarding study inclusion.

As this remains an emerging field of study, available literature is changing rapidly, hence conference abstracts were deemed acceptable for inclusion for review.^37^ Authors of conference abstracts were contacted to request further information and raw data. Regardless of the form, such as poster or presentation slides, the information was included to ensure a more detailed critique of included literature.

Records that progressed to the full-text review stage underwent two final assessments. Records were compared to allow exclusion of conference abstracts that were later published and were therefore duplicates despite different titles and years of publication. During the final screening, the first author also examined all references cited by the included records to ensure an adequate systematic review search had been conducted. Any records which met inclusion criteria during this stage of reviewing the reference lists were added to be reviewed. Records deemed to meet eligibility criteria on full-text review were included in the systematic review and were then reviewed and critiqued by the primary author as per the PRISMA guidelines.^29^

### Data collection items and process

The first author extracted data from all the included studies in the systematic review into the data extraction tool, which was developed using Microsoft Excel. Each study represented a row, with the various data categories and their sub-headings in columns. The data categories included record descriptors, study features, education curriculum features and outcomes. Record descriptors included title, author, year, journal, and record type. Study features included study design, aim/hypothesis, location, participants, sample size and study period. Education curriculum features included the education curriculum focus and details. Outcomes included key outcome, key result, other outcomes, other results and conclusion.

A simplified classification system for the education curriculum focus was developed following identification of the main goals of education by review of available literature. These included inhalational agent choice, lowering fresh gas flow, encouraging use of total intravenous anaesthesia (TIVA), switching off volatile anaesthetics on patient transfer, or a combination of these.

### Study risk of bias assessment

Critical appraisal of the included studies was assessed through use of the QualSyst appraisal tool.^38^ The QualSyst tool provided a systematic, standardised approach to appraise each study critically and allowed comparison of study methodological quality. The QualSyst checklist for assessing the quality of quantitative studies includes 14 criteria.^38^ The first and second authors independently appraised all included manuscripts using this checklist. The reviewers’ scores for each record were then compared. Disagreements were solved by discussion until consensus agreement within 5% was achieved and the first author’s score was used. The QualSyst score was used to interpret methodological quality and risk of bias by categorising them into the following categories:

- ≥80%: Strong methodological quality, low risk of bias;
- 55–79%: Adequate methodological quality, some concerns for risk of bias;
- <55%: Poor methodological quality, high risk of bias.

### Data synthesis and analysis

For data synthesis, the collected data were viewed in table form in Microsoft Excel (available on request). Descriptive statistics included counts of studies (*n*), proportion (%), median and interquartile range (IQR). The country of origin of the study, year published, study design, study period, and study focus were converted into graphical form to aid analysis. Due to variability among the different studies’ educational features and measured outcomes, they were analysed using template analysis, a style of thematic analysis.^39^ It is a clear, systematic, flexible approach used to simplify, categorise, compare, and analyse data.^39^ An initial set of codes was created by the primary author based on the background literature, and then text within each record was categorised into these codes. At this stage, if there was no suitable code to classify an important section of text, a further code was created. After identification of themes, educational design was simplified and classified as either lectures, emails, conversation forums, visual prompts, or a combination of these methods. Conversation forums consisted of meetings, email discussion forums and face-to-face conversations. Visual prompts included posters, vaporiser labels, and prompt cards. After identification of themes, measured outcomes related to clinical practice were simplified and classified as either utilising inhalational anaesthetic agent purchasing records or clinical usage data. Due to the variability among included studies’ designs and outcomes, meta-analysis, effect measures and certainty assessment were not feasible.

## Results

### Study selection

**Figure 1.**
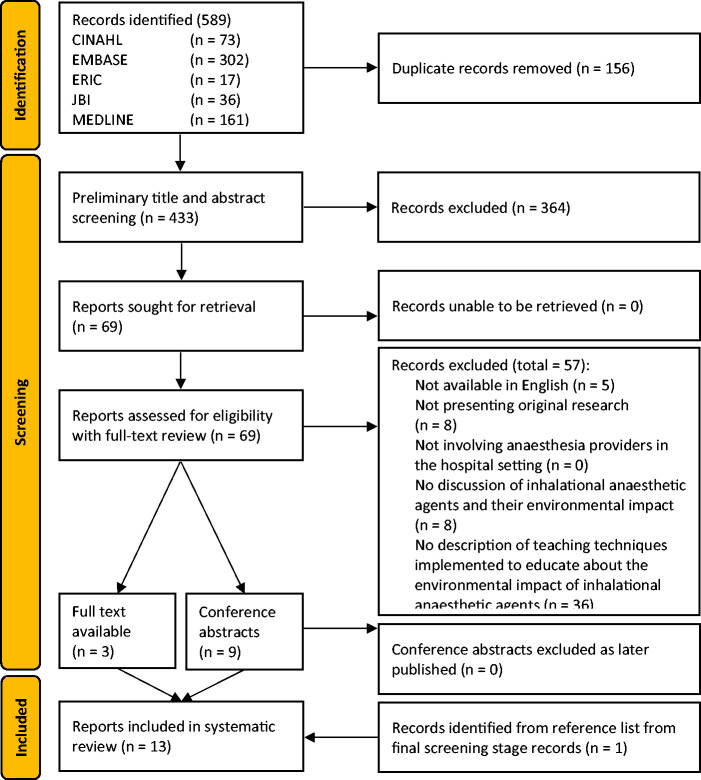
Preferred reporting items for systematic reviews and meta-analyses (PRISMA) diagram of study selection.

Initially, 589 records were identified before removal of 156 duplicates, with 433 records proceeding to preliminary title and abstract screening (Figure 1). A total of 69 records advanced to full-text review. At this stage, 57 records were excluded, with the most common reason for exclusion being records having no description of teaching methods about the environmental impact of inhalational anaesthetic agents (Supplementary Table 2). One record was identified through review of included studies’ references, leaving a total of 13 manuscripts^40–52^ for data extraction and critical appraisal.

### Study characteristics

Of the included studies, three were journal articles and 10 were conference abstracts (Table 2). Three of the 10 conference abstract authors responded to the email request for further information and raw data.^40,45,47^ All 13 were quantitative studies, in which 12 (92%) were pre–post interventional studies and one (8%) was a combination of prospective study with ongoing intervention and a cross-sectional survey.

**Table 2.** Systematic review record descriptors and study features.

Record descriptors	Study features
*Benness and Doane, 2021^40^Conference abstract	Pre–post intervention study. Australia. Single hospital. Nine-month study period.
Burrell, 2018^41^Journal article	Pre–post intervention study. New Zealand. Multiple hospitals (two). Three-year study period.
Danby et al, 2018^42^Conference abstract	Pre–post intervention study. England. Single hospital. Three-month study period.
Danby et al, 2018^43^Conference abstract	Pre–post intervention study. England. Single hospital. Three-month study period.
Glenski and Levine, 2020^44^Journal article	Pre–post intervention study. USA. Single paediatric hospital. Nine-month study period.
*Hickman and Molyneux, 2019^45^Conference abstract	Pre–post intervention study. England. Multiple hospitals. 14-Month study period.
Jameson and Young, 2021^46^Conference abstract	Pre–post intervention study. Scotland. Multiple hospitals (two). Study period not specified.
*Jani and Kalla, 2018^47^Conference abstract	Pre–post intervention study. England. Single hospital. Six-month study period.
Kennedy et al, 2022^48^Conference abstract	Prospective study with ongoing intervention + cross-sectional survey. Scotland. Multiple hospitals within the Scotland National Health Service. Three-year study period.
Kirkman et al, 2021^49^Conference abstract	Pre–post intervention study. England. Single hospital. Six-month study period.
Saadat et al, 2021^50^Conference abstract	Pre–post intervention study. Wales. Multiple hospitals (six). Study period not specified.
Sidana et al, 2011^51^Conference abstract	Pre–post intervention study. England. Single hospital. 10-Month study period.
Zuegge et al, 2019^52^Journal article	Pre–post intervention study. USA. Multiple hospitals (six, including one paediatric). Five-year study period.

* Denotes conference abstracts that provided further information.

In total, 69% (*n* = 9) were conducted in the UK, 15% (*n* = 2) were conducted in North America and 15% (*n* = 2) were conducted in Australia or New Zealand. The earliest study included in the systematic review was from 2011.^51^ No included studies were published between 2011 and 2018. The second earliest study included in the systematic review was published in 2018.^43^ The most recent study included was published in early 2022.^48^

Although not always specifically defined, the education session participants were suggested to be anaesthetists for all included studies. Seven studies were conducted at a single hospital^40,42–44,47,49,51^ and six studies were conducted across multiple hospitals,^41,45,46,48,50,52^ including two that incorporated paediatric hospitals (Table 2).^44,52^ The majority of the studies did not specify the specific services offered by the hospital. The study period of the interventional studies varied from 3 months to 5 years, with a median of 9 months (IQR 19 months).

### Risk of bias in studies

Results from the QualSyst tool for quantitative studies (Table 3) indicated that three studies were of adequate methodological quality with some concerns for risk of bias,^40,44,52^ and 10 studies were rated of poor methodological quality with a high risk of bias,^41–43,45–51^ all of which were conference abstracts. Despite the difficulty of assessing risk of bias of conference abstracts and therefore their associated high risk of bias, these were retained in the systematic review due to the emerging nature of this research topic.

**Table 3.** QualSyst score breakdown for included records.

Record descriptors	Checklist for quantitative studies (Y = 2, Partial = 1, No = 0 or N/A)														
	1. Question	2. Study design	3. Method of subject	4. Subject	5. Random allocation	6. Blinding of investigators	7. Blinding of subjects	8. Outcome	9. Sample size	10. Analytic methods	11. Variance	12. Confounding	13. Results	14. Conclusions	Total score (%)
*Benness and Doane, 2021^40^	2	2	2	1	0	0	0	2	2	1	0	1	2	2	61%
Burrell, 2018^41^	1	2	2	2	0	0	0	1	2	1	0	0	1	1	46%
Danby et al, 2018^42^	1	1	2	1	0	0	0	2	2	1	0	0	1	1	43%
Danby et al, 2018^43^	1	1	2	1	0	0	0	1	2	1	0	0	1	1	39%
Glenski and Levine, 2020^44^	2	2	2	2	0	0	0	2	2	2	2	1	2	2	75%
*Hickman and Molyneux, 2019^45^	1	1	2	1	0	0	0	2	2	1	0	0	1	2	46%
Jameson and Young, 2021^46^	2	2	2	1	0	0	0	2	1	1	0	0	1	2	50%
*Jani and Kalla, 2018^47^	2	2	2	1	0	0	0	2	1	1	0	0	2	2	54%
Kennedy et al, 2022^48^	2	2	1	1	0	0	0	1	2	1	0	1	1	2	50%
Kirkman et al, 2021^49^	1	1	2	1	0	0	0	2	2	1	0	0	1	2	46%
Saadat et al, 2021^50^	2	2	2	1	0	0	0	1	2	1	0	0	1	2	50%
Sidana et al, 2011^51^	1	1	2	1	0	0	0	2	2	1	0	0	1	2	46%
Zuegge et al, 2019^52^	2	2	2	2	0	0	0	2	2	1	1	1	2	1	64%

*Denotes conference abstracts that provided further information.

### Results of individual studies

Individual study features can be seen in Table 2. Each study’s education curricula focus and details, alongside other interventions for each study were extracted (Table 4). The measured outcomes for each individual study and a summary of key results can be seen in Table 5.

**Table 4.** Systematic review education curriculum features and other interventions.

Record	Education curriculum focus	Education curriculum details	Other interventions
*Benness and Doane, 2021^40^	Low FGF, inhalation agent choice & use of TIVA.	Departmental presentation (lecture), followed by departmental newsletters (emails) and placement of educational posters across targeted areas of the operating theatre complex (visual prompt—posters).	Nil.
Burrell, 2018^41^	Low FGF, inhalation agent choice & use of TIVA.	Reports education with no specifications on form (presumably lecture), published monthly audit data in graphical form via email (emails), and created conversation forums (online via emails).	Changed to low FGF machines at beginning of study & removed desflurane as default vaporiser during study.
Danby et al, 2018^42^	Inhalation agent choice.	Presented initial audit findings at departmental audit day (lecture).	Introduced new CO_2_ absorber into practice.
Danby et al, 2018^43^	Inhalation agent choice.	Departmental presentation (lecture).	Nil.
Glenski and Levine, 2020^44^	Low FGF.	Departmental presentations (lectures), departmental emails (emails), and the educational focus was regularly discussed at daily morning meetings (conversation forums).	Changed default programme on machine to low FGF.
*Hickman and Molyneux, 2019^45^	Inhalation agent choice.	Prompt cards containing facts regarding volatiles and the environment were placed on top of all anaesthetic machines with interactive anagrams with prizes (visual prompts).	Nil.
Jameson and Young, 2021^46^	Low FGF.	Reports an educational presentation (lecture) and email reminders (emails).	Nil.
*Jani and Kalla, 2018^47^	Low FGF & switching off gas on transfer.	Audit findings were shared with the department (lecture), sent email reminders (emails), and displayed visual prompts on theatre doors and anaesthetic machines (posters).	Education involved financial aspects alongside environmental impacts.
Kennedy et al, 2022^48^	Low FGF & inhalation agent choice.	Reports promoting environmentally stable anaesthesia through spreading the message through the Scottish Environmental Anaesthesia Group (conversation forum).	Nil.
Kirkman et al, 2021^49^	Inhalation agent choice.	Monthly clinical governance meeting presentations (lectures) and educational posters were displayed in anaesthetic rooms (visual prompts).	Removed desflurane as default vaporiser during study.
Saadat et al, 2021^50^	Low FGF, inhalation agent choice & use of TIVA	Educational presentations (lectures) and posters (visual prompts).	Nil.
Sidana et al, 2011^51^	Low FGF.	Initial audit data was presented at departmental meeting (lecture) and then low flow cards were designed and displayed on anaesthetic machines (visual prompts).	Nil.
Zuegge et al, 2019^52^	Low FGF & inhalation agent choice.	A new employee training presentation was implemented alongside volatile education being incorporated into annual waste reduction and green practices lecture (lectures), communications were sent every few months (emails), efforts were made via relationship building and one-on-one conversations (conversation forum), and vaporiser labels were developed with educational content (visual prompts).	Nil.

* Denotes conference abstracts that provided further information.

FGF: fresh gas flow; TIVA: total intravenous anaesthesia; CO_2_: carbon dioxide.

**Table 5.** Systematic review of measured outcomes and summary of key results.

Record	Measured outcome	Summary of key results
*Benness and Doane, 2021^40^	Carbon footprint of inhalation anaesthetic agents through measuring purchasing records and total number of anaesthetics performed.	↓ desflurane usage, sevoflurane usage, inhalation agent spending and CO_2_e.
Burrell, 2018^41^	Carbon footprint per minute of inhalational anaesthetic through measuring agent purchasing records and recording anaesthetic time.	↓ desflurane usage and CO_2_e.= sevoflurane usage and isoflurane usage.
Danby et al., 2018^42^	Carbon footprint of inhalational anaesthetic agents through measuring duration of anaesthetic, choice of inhalation agent and volume used.	↓ desflurane usage, sevoflurane usage and CO_2_e.↑ isoflurane usage.
Danby et al., 2018^43^	Carbon footprint of anaesthetic agents through measuring choice of anaesthetic, choice and duration of inhalational agent and volume used.	↓ desflurane usage and CO_2_e.↑ isoflurane usage.= sevoflurane usage and TIVA.
Glenski and Levine, 2020^44^	Carbon footprint through tracking sevoflurane purchasing records and total number of anaesthetics performed.	↓ sevoflurane usage and CO_2_e.
*Hickman and Molyneux, 2019^45^	Carbon footprint of desflurane through measuring purchasing records.	↓CO_2_e.
Jameson and Young, 2021^46^	Location of anaesthesia, initial FGF rate and time to commencing low flow anaesthesia.	↓ induction FGF rate.↑ use of low-flow anaesthesia.
*Jani and Kalla, 2018^47^	FGF rate and whether or not anaesthetic gas was turned off on transfer.	↓ gas left on after transfer= /↑ FGF rates.
Kennedy et al., 2022^48^	Carbon footprint of inhalation anaesthetic agents through measuring purchasing records per quarter per head of catchment population.	↓ desflurane usage and CO_2_e (reported without statistics).
Kirkman et al., 2021^49^	Carbon footprint of inhalational anaesthetic agents through measuring purchasing records and total number of anaesthetics performed.	↓ desflurane usage, inhalational anaesthetic spending and CO_2_e.↑ sevoflurane usage.
Saadat et al., 2021^50^	Carbon footprint of inhalational anaesthetic agents through measuring inhalational anaesthetic usage. Also calculated costs.	↓ inhalational anaesthetic spending and CO_2_e.
Sidana et al., 2011^51^	Volatile agent choice, carrier gas choice, FGF rate, calculated volatile anaesthetic agent cost, and total number of anaesthetics performed.	↓ anaesthetic agent spending.↑ use of low-flow anaesthesia.
Zuegge et al., 2019^52^	Carbon footprint of inhalational anaesthetic agents through purchasing records and total number of anaesthetics performed.	↓ desflurane usage, inhalational anaesthetic spending and CO_2_e.↑ sevoflurane usage= isoflurane usage.

* Denotes conference abstracts that provided further information.

CO_2_e: carbon dioxide equivalent; TIVA: total intravenous anaesthesia; FGF: fresh gas flow.

### Results of syntheses

#### Focuses of education curricula to optimise the environmental impact of inhaled anaesthetic agents

**Figure 2.**
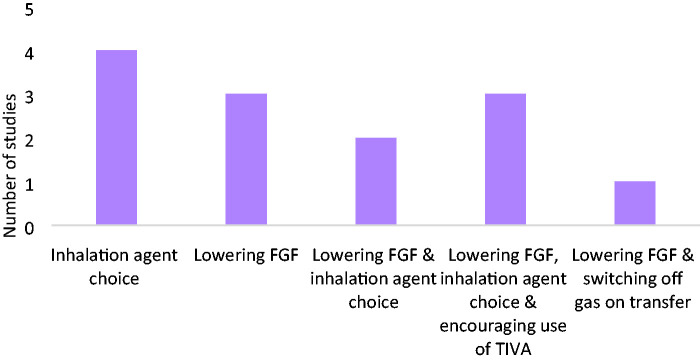
Education strategies for promoting behavioural change by anaesthetists in regard to the environmental impact of anaesthesia. FGF: fresh gas flow; TIVA: total intravenous anaesthesia.

Optimisation of the environmental impact of inhalational anaesthetic agents was targeted through focusing on multiple approaches (Table 4 and Figure 2). Approximately half (54%, *n* = 7) concentrated on a single approach with the remaining (46%, *n* = 6) utilising a multimodal approach. Of the 13 studies, 31% (*n* = 4) focused on optimising the environmental impact of inhalational anaesthetic agents through education encouraging alterations of inhalational agent choice as a single approach, whereas 23% (*n* = 3) focused on education encouraging lowering the fresh gas flow used as a single approach. Meanwhile, 15% (*n* = 2) focused on education regarding both lowering fresh gas flow and inhalational agent choice. In addition to targeting lowering fresh gas flow and inhalational agent choice, 23% (*n* = 3) of studies also focused on education about utilisation of TIVA. In addition to targeting lowering fresh gas flow, 8% (*n* = 1) also focused on education about the importance of switching off gas on transfer out of the anaesthetic room. Overall, education curricula focused on inhalational agent choice (69%), lowering the fresh gas flow during maintenance anaesthesia (69%), encouraging alternatives such as TIVA (23%) and/or switching off the gas on transfer (8%).

#### Education techniques to optimise the environmental impact of inhalational anaesthetic agents

**Figure 3.**
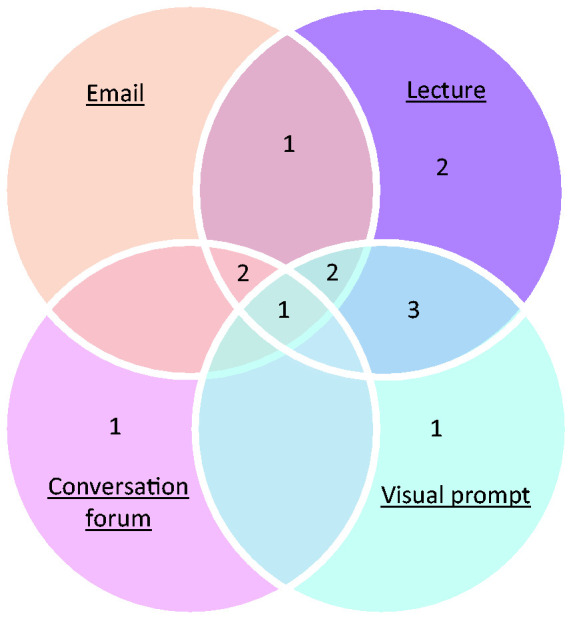
Education modalities used in studies on promoting behavioural change by anaesthetists in regard to the environmental impact of anaesthesia.

Didactic lectures were the most common education modality with 85% (*n* = 11) utilising this approach (Table 4 and Figure 3). Visual prompts, including posters, vaporiser labels and prompt cards, were used in 54% (*n* = 7) of studies. Electronic mail communication was used in 46% (*n* = 6). Conversation forums, including meetings, email discussion forums and face-to-face conversations, were the least common technique with 31% (*n* = 4) utilising this approach. Overall, 69% (*n* = 9) used multiple techniques.

#### Methods of measuring the effect of an education curriculum on the environmental impact of anaesthetic agents

Tracking environmental impact was most commonly done by audit of purchasing records and was performed by 54% (*n* = 7) of studies (Table 5). Of these, five studies tracked purchasing records for multiple anaesthetic gases,^40,41,48,49,52^ whereas two studies collected data for a single agent.^44,45^ The other 46% (*n* = 6) of studies collected detailed data about inhalational anaesthetic agent usage, such as anaesthetic time, agent used and flow rates.

#### Change in anaesthesia carbon footprint through education encouraging change in anaesthetist behaviour

The majority of included studies (92%, *n* = 12) reported that educational interventions correlated with lower inhalational anaesthetic agent use (Table 5). The remaining study discussed a partial transition in anaesthetist behaviour following education about low fresh gas flow and turning off the gas on transfer through a single lecture, visual prompts, and email reminders over a 6-month period.^47^ Data were collected through questionnaires and showed an improvement in the gas being switched off on transfer, but did not show an improvement in low fresh gas flow utilised.

Of note, four of the included studies implemented concurrent non-educational interventions during their study periods. Danby et al.^42^ introduced a CO_2_ absorber to anaesthetic machines at the same time as the education curriculum, while Glenski and Levine^44^ changed the default programme on the anaesthetic machine to a low-flow anaesthesia programme. Kirkman et al.^49^ changed their anaesthetic machines to a model with a preference for low fresh gas flows at the beginning of the study period, and midway through two study periods^41,49^ desflurane was removed as the default agent. Meanwhile, Burrell^41^ provided equivalent education on both the environmental and financial impact of anaesthetic agents.

## Discussion

All studies in the systematic review reported on interventions that were associated with behaviour change that lowers the environmental impact of anaesthetic agents, with all but one study^47^ demonstrating this for all measured outcomes. It is presumed that the change in anaesthetic usage was due to the education curriculum causing behavioural change of individual anaesthetists. Each study achieved change within their respective anaesthetic departments through different key focuses and educational curricula. This systematic review suggests that multiple methods of educating anaesthetists about the impact of inhalational anaesthetic agents on the environment are impactful in affecting behaviour change, resulting in a reduced carbon footprint. This reinforces the notion that any education is beneficial as a means of disseminating updated information and challenging rigid practice protocols and habits.

The four focuses for education categorised in this systematic review were inhalational agent choice, lowering fresh gas flow, encouraging use of TIVA and switching off gas on transfer. These align with current guidelines and recommendations.^18–22^ The World Federation of Societies of Anaesthesiologists reported ‘[a]naesthesia providers should use environmentally preferable medications and equipment when clinically safe to do so’.^19^^(p. 204)^ They reached consensus and reported ‘[a]naesthesia providers should always consider how they can safely reduce the amount of drugs, equipment, energy and water used in their practice, for environmental reasons’.^19^^(p. 204)^

All included studies reviewed purchasing records or detailed anaesthetic usage as their measured outcomes. The outcome was measured both before and after an education curriculum was implemented to assess for change. The majority of studies measured purchasing records for multiple agents. When optimising the environmental impact of anaesthetic agents, coincidentally there are economic benefits. Desflurane is more expensive than the alternative volatile agent choices.^52^ Therefore, shifting away from the use of desflurane results in a decrease in carbon footprint alongside cost savings. Utilising closed-circuit breathing systems which reuse exhaled anaesthetic gases reduces the amount of fresh anaesthetic gas flow required, and therefore decreases the overall emissions and cost. Similar gains are achieved by switching off the gas on patient transfer. Shifting away from inhalational anaesthetic agents and towards alternatives such as TIVA also has economic benefits, as propofol is significantly cheaper.^53^ Financial benefits were reported as part of the teaching curriculum in just one included study.^47^ Fiscal responsibility has likely been the main driver for historical change in anaesthetic usage. Both decreasing costs and carbon footprint while maintaining patient safety in combination have the potential to be great motivators for behavioural change particularly at a departmental and organisational level.

### Limitations of included studies

The first limitation of the included studies was low Qualsyst scores, which is likely due to the type of article. This is an emerging field of study and there is therefore limited available evidence, leading to the inclusion of 10 conference abstracts. Although further information about the studies was requested, only three authors responded. An author is responsible for communicating their research findings within the constraints of the publishing guidelines. As conference abstracts have a strict word limit, they are inherently missing sufficient detail, especially with regard to results. In addition, conference abstracts are not always peer reviewed. As a consequence, there is a significant reporting bias and a lower QualSyst score, denoting increased risk of bias. For an emerging field of knowledge, this may be expected. Collation of available evidence by this systematic review aims to solidify the foundation on which future researchers can build.

A second limitation was that the studies reviewed purchasing records or detailed anaesthetic usage as their measured outcomes. Both of these outcomes are inherently associated with limitations and bias. Using purchasing records as a surrogate for inhalational anaesthetic agent usage has the benefit of allowing large sample sizes as they capture the purchasing records for the entire hospital, although there are multiple factors that influence purchasing records including hospital-wide patient numbers, inhalational anaesthetic use, and purchasing price. Through utilising hospital purchasing records, the data encapsulates hospital-wide use of inhalational anaesthetic agents including the obstetric, dental and emergency departments if present within the hospital. These departments did not participate in the education sessions provided, likely diluting the positive impacts of teaching sessions. This fact was not recognised in most included literature. To allow for generalisation, some studies compared it with either catchment population, total anaesthetic case numbers, or anaesthetic time. Measuring anaesthetic usage can involve extracting data from the anaesthetic machine, extracting data from electronic anaesthetic records, employing a third party to collect data, or through an anaesthetist completing questionnaires. The anaesthetist would be aware when a third party was collecting data in the theatre on their anaesthetic practice. This may introduce an observer bias as the anaesthetist may change their regular practice while being observed.^54^ An anaesthetist completing a questionnaire on their own practice may introduce a response bias.^55^

### Limitations of the review process

Confounding factors identified by studies were generally not accounted for when assessing for behavioural and institutional change. Some included studies implemented multiple interventions at the same time. Multiple interventions may be required to encourage behavioural change.^56^ An inability to conduct a meta-analysis was also an inherent limitation due to the variation among the outcomes of the studies included and limited accessible methodological information. As the educational methods were unable to be compared this review cannot comment on the relative efficacy of the educational methods.

### Multilevel education recommendations

Anaesthetic departments should implement education curricula on the topic of the environmental impact of anaesthesia (greener anaesthesia), and anaesthetic colleges should incorporate education on greener anaesthesia into formal anaesthetic training curricula. This education should focus on the most relevant key approaches to creating greener anaesthesia within the department. Ideally, multimodal and continuous education would be delivered, but other less intensive formats of education are still effective. In 2022, the World Federation of Societies of Anaesthesiologists collated knowledge of greener anaesthetic practice around the world, and created guidelines and recommendations that can be implemented internationally.^19^ Institutions should introduce policies based on these guidelines. These should be adhered to, in order to create change towards friendlier anaesthesia.

### Implications for future research

It is presumed that education curricula can result in change in anaesthetic usage through behavioural change of individual anaesthetists; however, further research investigating this relationship is required. Further research in the field needs to be conducted, ideally with a standardised approach to allow for meta-analysis.^57^ This would allow direct comparison of the outcomes to assess which educational techniques are the most effective. This systematic review suggests that a possible standardised approach to purchasing records is to compare purchasing records of the anaesthetic department with anaesthetic time, or anaesthetic case numbers. This could be done on a random sample and extrapolated to the entire study sample. Due to this being a rapidly emerging topic, this article could form the basis for a living systematic review.^58^

## Conclusion

Despite the paucity and lack of quality of available data, all available data appear to be consistent in suggesting that single education sessions as well as multifocused, multimodal education curricula on greener anaesthesia are beneficial in promoting behavioural change. The education curriculum approaches in this review focused on shifting away from desflurane and nitrous oxide usage, reducing inhalational anaesthetic agent waste, use of low-flow anaesthesia and/or refraining from use of inhalational anaesthesia completely in preference of alternatives such as TIVA.

## Supplemental Material

sj-pdf-1-aic-10.1177_0310057X241263113 - Supplemental material for Promoting behavioural change by educating anaesthetists about the environmental impact of inhalational anaesthetic agents: A systematic review Supplemental material, sj-pdf-1-aic-10.1177_0310057X241263113 for Promoting behavioural change by educating anaesthetists about the environmental impact of inhalational anaesthetic agents: A systematic review by C Nolan, Michael J Hoskins, Bríd Phillips and Kiah L Evans in Anaesthesia and Intensive Care
