# Supplementary material for: Promoting behavioural change by educating anaesthetists about the environmental impact of inhalational anaesthetic agents: A systematic review
Source: Anaesth Intensive Care. 2024 Aug 30;52(6):356–68. doi: 10.1177/0310057X241263113 (PMC11531086; doi:10.1177/0310057X241263113)
Supplement: sj-pdf-1-aic-10.1177_0310057X241263113 - Supplemental material for Promoting behavioural change by educating anaesthetists about the environmental impact of inhalational anaesthetic agents: A systematic review [file sj-pdf-1-aic-10.1177_0310057X241263113.pdf]

Supplemental Table 1. Search strategy for systematic review.

Database	Search terms	Number of records
CINAHL Plus	educat* or train* or learn* or instruct* or teach* or staff development or (MH "Education+") or (MH "Teaching+") or (MH "Staff Development") or (MH "Learning+")<br>AND<br>carbon footprint* or climate change or emission* or greenhouse or pollut* or global warming or (MM "Climate Change+") or (MM "Greenhouse Effect") or (MH "Greenhouse Gases") or (MM "Carbon Footprint")<br>AND<br>an#esth* or desflurane or sevoflurane or isoflurane or nitrous oxide or Entonox or (MH "Anesthetics+") or (MH "Anesthesia+") or (MH "Anesthesia Induction") or (MH "Anesthesia and Analgesia+") or (MH "Desflurane") or (MH "Sevoflurane") or (MH "Isoflurane") or (MH "Nitrous Oxide")	73
Embase	educat*.tw. or train*.tw. or learn*.tw. or instruct*.tw. or teach*.tw. or staff development.tw. or clinical education/ or medical education/ or health education/ or continuing education/ or doctoral education/ or	302

	residency education/ or adult education/ or nurse anesthesia education/ or education/ or learning environment/ or lifelong learning/ or learning/ or teaching/ or teaching round/ or teaching hospital/ or in service training/<br>AND<br>carbon footprint*.tw. or climate change.tw. or emission*.tw. or greenhouse.tw. or pollut*.tw. or global warming.tw. or (environment* adj3 (chang* or effect* or warm* or sustain*)).tw. or carbon footprint/ or greenhouse effect/or greenhouse gas/ or global climate/ or climate change/ or climate warming/<br>AND<br>an?esth*.tw. or desflurane.tw. or isoflurane.tw. or sevoflurane.tw. or nitrous oxide.tw. or entonox.tw. or anesthetic agent/ or anesthetist/ or anesthesiologist/ or anesthesiology/ or anesthesia/ or general anesthesia/ or inhalation anesthesia/ or intravenous anesthesia/ or anesthesia gas machine/ or anesthesia induction/ or closed circuit anesthesia/ or low flow anesthesia/ or isoflurane/ or sevoflurane/ or desflurane/ or nitrous oxide/	
ERIC	noft(educat*) or noft(train*) or noft(learn*) or noft(instruct*) or noft(teach) or noft(staff development*) or MAINSUBJECT.EXACT("Inservice Education") or MAINSUBJECT.EXACT("Health Education") or	17

	MAINSUBJECT.EXACT(“Medical Education”) or MAINSUBJECT.EXACT(“Lifelong Learning”) or MAINSUBJECT.EXACT(“Adult Learning”) or MAINSUBJECT.EXACT(“Environmental Education”)<br>AND<br>noft(carbon footprint*) or noft(climate change*) or noft(emission*) or noft(greenhouse) or noft(pollut) or noft(global warming) or noft(environment* near/3 (chang* or effect* or warm* or sustain*)) or MAINSUBJECT.EXACT(“Climate”) or MAINSUBJECT.EXACT(“Pollution”) or MAINSUBJECT.EXACT(“Environment”) or MAINSUBJECT.EXACT(“Air Pollution”) or MAINSUBJECT.EXACT(“Climate Control”)<br>AND<br>noft(an*esth*) or noft(desflurane) or noft(isoflurane) or noft(sevoflurane) or noft(nitrous oxide) or noft(Entonox) or MAINSUBJECT.EXACT(“Anesthesiology”) or MAINSUBJECT.EXACT(“Surgery”)	
Joanna Briggs Institute	educat*.tw. or train*.tw. or learn*.tw. or instruct*.tw. or teach*.tw. or staff development.tw.<br>AND<br>climate change.tw. or emission*.tw. or greenhouse.tw. or pollut*.tw. or global warming.tw. or (environment* adj3 (chang* or effect* or warm* or sustain*)).tw.<br>AND	36

	an?esth*.tw. or desflurane.tw. or isoflurane.tw. or sevoflurane.tw. or nitrous oxide.tw. or entonox.tw.	
Medline	educat*.tw. or train*.tw. or learn*.tw. or instruct*.tw. or teach*.tw. or staff development.tw. or education.fs. or education/ or teaching/ or inservice training/ or staff development/ or exp self-directed learning as topic/ or education, medical, continuing/ or education, continuing/ or education, professional/ or education, medical, graduate/ or learning/<br>AND<br>carbon footprint*.tw. or climate change.tw. or emission*.tw. or greenhouse.tw. or pollut*.tw. or global warming.tw. or (environment* adj3 (chang* or effect* or warm* or sustain*)).tw. or climate change/ or global warming/ or greenhouse effect/ or ozone depletion/ or greenhouse gases/ or carbon footprint/<br>AND<br>an?esth*.tw. or desflurane.tw. or isoflurane.tw. or sevoflurane.tw. or nitrous oxide.tw. or 4ntonox.tw. or anesthesia/ or anesthesia, general/ or exp anesthesia, inhalation/ or anesthesia, intravenous/ or anesthetics/ or exp anesthetics, general/ or anesthesiologists/ or anesthesia, closed-circuit/ or desflurane/ or isoflurane/ or sevoflurane/ or nitrous oxide/	161

Supplemental Table 2. Excluded records at full text review with reason for exclusion.

Record	Reason for record exclusion
A compromise for closed system anesthesia (Gorsky, 1978).[1]	No description of teaching techniques implemented to educate learners about the environmental impact of inhalational anaesthetic agents.
A national survey on attitudes and barriers on recycling and environmental sustainability efforts among Canadian anesthesiologists: an opportunity for knowledge translation (Petre, 2019).[2]	No description of teaching techniques implemented to educate learners about the environmental impact of inhalational anaesthetic agents.
A new role for anaesthetists in environmentally-sustainable healthcare (Shelton, 2019).[3]	Not presenting original research.
A survey of the American Society of Anesthesiologists regarding environmental attitudes, knowledge, and organization (Ard, 2016).[4]	No description of teaching techniques implemented to educate learners about the environmental impact of inhalational anaesthetic agents.
A survey of the anesthesia scavenging systems in a teaching hospital (Soontranan, 2002).[5]	No description of teaching techniques implemented to educate learners about the environmental impact of inhalational anaesthetic agents.

Ambient isoflurane pollution and isoflurane consumption during intensive care unit sedation with the Anesthetic Conserving Device (Sackey, 2005).[6]	No description of teaching techniques implemented to educate learners about the environmental impact of inhalational anaesthetic agents.
An audit of utility rates of low-flow anaesthesia in relation to provider experience and its economic implications in an acute general hospital (Hoa, 2018).[7]	No description of teaching techniques implemented to educate learners about the environmental impact of inhalational anaesthetic agents.
Anaesthetic waste disposal (Jaladi, 2011).[8]	No discussion of inhalational anaesthetic agents and their environmental impact.
Anesthesia and sedation for pediatric patients undergoing procedures outside of the operating room (Beebe, 2000).[9]	No description of teaching techniques implemented to educate learners about the environmental impact of inhalational anaesthetic agents.
Anesthesia environmental sustainability programs-a survey of Canadian department chiefs and residency program directors (Petre, 2020).[10]	No description of teaching techniques implemented to educate learners about the environmental impact of inhalational anaesthetic agents.
Anesthesiologists in times of disaster: a rich history, a busy future (Raiten, 2021).[11]	No description of teaching techniques implemented to educate learners about the environmental impact of inhalational anaesthetic agents.

Attitudes towards use of nitrous oxide amongst trainee anaesthetists (Patel, 2012).[12]	No description of teaching techniques implemented to educate learners about the environmental impact of inhalational anaesthetic agents.
Audit of efficiency of volatile use (Sadler, 2016).[13]	No description of teaching techniques implemented to educate learners about the environmental impact of inhalational anaesthetic agents.
Barriers to and opportunities for recycling, reducing and reusing in Canadian academic anesthesia departments (Petre, 2017).[14]	No description of teaching techniques implemented to educate learners about the environmental impact of inhalational anaesthetic agents.
Changes are afoot (Webster, 2015).[15]	Not presenting original research.
Closed circuit anesthesia: A perspective in clinical practice [Japanese] (Morita, 1994).[16]	Not available in English.
Coordinated eco-leadership can unite clinicians towards net zero (Davies, 2021).[17]	Not presenting original research.
Delivering medicines optimisation in paediatric theatres (Brooks, 2019).[18]	No description of teaching techniques implemented to educate learners about the environmental impact of inhalational anaesthetic agents.

Ecological sustainability in anaesthesiology and intensive care medicine. A DGAI and BDA position paper with specific recommendations [German] (Schuster, 2020).[19]	No description of teaching techniques implemented to educate learners about the environmental impact of inhalational anaesthetic agents.
Education for sustainable health care: from learning to professional practice (Huss, 2020).[20]	No description of teaching techniques implemented to educate learners about the environmental impact of inhalational anaesthetic agents.
Efficacy of simple scavenging system applied for volatile-based, long-term ICU sedation (Wasowicz, 2014).[21]	No description of teaching techniques implemented to educate learners about the environmental impact of inhalational anaesthetic agents.
Eighteen years of exploring patterns of anaesthetic gas flows with more recent data on CO_2 GWP footprint (Kennedy, 2020).[22]	No description of teaching techniques implemented to educate learners about the environmental impact of inhalational anaesthetic agents.
Empowering health-care learners to take action towards embedding environmental sustainability into health-care systems (Luo, 2021).[23]	No description of teaching techniques implemented to educate learners about the environmental impact of inhalational anaesthetic agents.
Environmental and economic impact of using increased fresh gas flow to reduce carbon dioxide absorbent consumption in the	No description of teaching techniques implemented to educate learners about the environmental impact of inhalational anaesthetic agents.

absence of inhalational anaesthetics (Zhong, 2020).[24]	
Environmental and occupational hazards of the anesthesia workplace (Kole, 1990).[25]	No description of teaching techniques implemented to educate learners about the environmental impact of inhalational anaesthetic agents.
Environmental sustainability in anaesthesia and critical care (McGain, 2020).[26]	Not presenting original research.
Evaluation of the environmental monitoring's effects on the anaesthetic gases concentrations in the operating theatres [Italian] (Albertini, 2008).[27]	Not available in English.
Gas Man is an in silico translational tool to improve care and reduce inhalation anesthetic waste, cost, and pollution (Feldman, 2021).[28]	No description of teaching techniques implemented to educate learners about the environmental impact of inhalational anaesthetic agents.
Go with the flow: A two-week low flow and closed circuit anesthesia elective for pediatric anesthesia clinical fellows: experience of the first two years (Navedo, 2012).[29]	No description of teaching techniques implemented to educate learners about the environmental impact of inhalational anaesthetic agents.
Greening the OR. Hospitals and Health Networks (Jarousse, 2015).[30]	Not presenting original research.

How low can you go? A survey of current practice as the first step towards sustainable anaesthesia in a district general hospital (Lusby, 2019).[31]	No description of teaching techniques implemented to educate learners about the environmental impact of inhalational anaesthetic agents.
Improving training in paediatric total intravenous anaesthesia using experience-based co-design methodology (Brooks, 2020).[32]	No discussion of inhalational anaesthetic agents and their environmental impact.
Inhaled Nitrous Oxide for Labor Analgesia (Starr, 2013).[33]	No description of teaching techniques implemented to educate learners about the environmental impact of inhalational anaesthetic agents.
Initiatives to broaden safety concerns in anaesthetic practice: the green operating room (Beloeil, 2021).[34]	No description of teaching techniques implemented to educate learners about the environmental impact of inhalational anaesthetic agents.
Minimal flow anaesthesia. A modern alternative for daily practice [German] (Spiess, 1980).[35]	Not available in English.
Myth and reality: A survey to determine anaesthetists' knowledge and attitudes governing low-flow anaesthesia (Sorrentino, 2011).[36]	No description of teaching techniques implemented to educate learners about the environmental impact of inhalational anaesthetic agents.

Nitrous oxide - 200 years of history, a splendid present, doubtful future? (Schneck, 2002).[37]	No description of teaching techniques implemented to educate learners about the environmental impact of inhalational anaesthetic agents.
Nitrous oxide pollution in operating theatres in relation to the type of leakage and the number of efficacious air exchanges per hour (Sartini, 2006).[38]	No description of teaching techniques implemented to educate learners about the environmental impact of inhalational anaesthetic agents.
Nitrous oxide: occupational hazards (Nnaji, 2021).[39]	Not presenting original research.
Operation clean up in Australian operating theatres - a quality improvement program by TRA2SH (Davies, 2021).[40]	No discussion of inhalational anaesthetic agents and their environmental impact.
Perioperative settings: recycling for waste reduction (Minooee, 2021).[41]	Not presenting original research.
Perioperative settings: reuse and waste reduction (Aginga, 2021).[42]	Not presenting original research.
Pollution of ambient air by volatile anesthetics: a comparison of 4 anesthetic management techniques (Barberio, 2006).[43]	No description of teaching techniques implemented to educate learners about the environmental impact of inhalational anaesthetic agents.

Pollution of operating theatres, flammable agents and other matters (Keen, 1980).[44]	No description of teaching techniques implemented to educate learners about the environmental impact of inhalational anaesthetic agents.
Principles of environmentally-sustainable anaesthesia: a global consensus statement from the World Federation of Societies of Anaesthesiologists (White, 2022).[45]	No description of teaching techniques implemented to educate learners about the environmental impact of inhalational anaesthetic agents.
Proposal of a health education program [Italian] (Signorini, 1992).[46]	Not available in English.
Recognising our carbon footprint in anaesthesia and the potential for improvements (MacKenzie, 2018).[47]	No description of teaching techniques implemented to educate learners about the environmental impact of inhalational anaesthetic agents.
Recycling and greener anaesthesia at Mid Essex Hospital NHS Trust: quality-improvement project (Jandu, 2015).[48]	No discussion of inhalational anaesthetic agents and their environmental impact.
Recycling in anaesthetic rooms: striving for a sustainable environment (Selvaraju, 2018).[49]	No discussion of inhalational anaesthetic agents and their environmental impact.
Reducing carbon emissions: one gas at a time (Whitehead, 2021).[50]	No description of teaching techniques implemented to educate learners about the environmental impact of inhalational anaesthetic agents.

Sustainability in general anaesthesia: attitudes and capability regarding inhalational gas versus total intravenous anaesthesia (Craven, 2021).[51]	No description of teaching techniques implemented to educate learners about the environmental impact of inhalational anaesthetic agents.
The future of anesthesiology: implications of the changing healthcare environment (Prielipp, 2016).[52]	No discussion of inhalational anaesthetic agents and their environmental impact.
The use of nitrous oxide in anaesthetic practice: a questionnaire survey (Henderson, 2002).[53]	No description of teaching techniques implemented to educate learners about the environmental impact of inhalational anaesthetic agents.
Towards greener anaesthesia: a project to reduce volatile anaesthetic use in a large teaching hospital (Campbell, 2021).[54]	No discussion of inhalational anaesthetic agents and their environmental impact.
Train healthcare professionals for environmental sustainability of health services (Baures, 2021).[55]	No discussion of inhalational anaesthetic agents and their environmental impact.
Training in hygiene for prevention of environmental pollution by anesthetic gases [Italian] (Citerio, 1985).[56]	Not available in English.
Use of volatile anaesthetic agents in anaesthesia: a survey of practice in France in 2012 (Benhamou, 2015).[57]	No description of teaching techniques implemented to educate learners about the environmental impact of inhalational anaesthetic agents.

REFERENCES

1. Gorsky B, Hall R, Redford J. A compromise for closed system anesthesia. *Anesth Analg*. 1978;57(1):18-24.

2. Petre M-A, Bahrey L, Levine M, van Rensburg A, Crawford M, Matava C. A national survey on attitudes and barriers on recycling and environmental sustainability efforts among Canadian anesthesiologists: an opportunity for knowledge translation. *Can J Anaesth*. 2019;66(3):272-286.

3. Shelton C, McBain S, Mortimer F, White S. A new role for anaesthetists in environmentally-sustainable healthcare. *Anaesthesia*. 2019;74(9):1091-1094.

4. Ard JL, Tobin K, Huncke T, Kline R, Ryan SM, Bell C. A survey of the American Society of Anesthesiologists regarding environmental attitudes, knowledge, and organization. *A & A Case Reports*. 2016;6(7):208-216.

5. Soontranan P, Lertakyamanee J, Somprakit P, Surachetpong S. A survey of the anesthesia scavenging systems in a teaching hospital. *Journal of the Medical Association of Thailand/Chotmaihet Thangphaet*. 2002;85:S824-9.

6. Sackey PV, Martling C-R, Nise G, Radell PJ. Ambient isoflurane pollution and isoflurane consumption during intensive care unit sedation with the Anesthetic Conserving Device. *Crit Care Med*. 2005;33(3):585-590.

7. Hoa L, Lye S, Wong M. An audit of utility rates of low-flow anaesthesia in relation to provider experience and its economic implications in an acute general hospital. 2018:48-48.

8. Jaladi S, Corner A. Anaesthetic waste disposal. *Br J Anaesth*. 2011;107(2):286-287.

9. Beebe D. Anesthesia and sedation for pediatric patients undergoing procedures outside of the operating room. *J Perianesth Nurs*. 2000;21

10. Petre M-A, Bahrey L, Levine M, van Rensburg A, Crawford M, Matava CT. Anesthesia environmental sustainability programs—a survey of Canadian department chiefs and residency program directors. *Can J Anaesth*. 2020;67(9):1190-1200.

11. Raiten J, Fleisher L. Anesthesiologists in times of disaster: a rich history, a busy future. *Anesthesiol Clin*. 2021;39(2):xi-xii.

12. Patel P, Patel N, Jani K. Attitudes towards use of nitrous oxide amongst trainee anaesthetists. *Anaesthesia*. 2012;67(2)

13. Sadler A, Ward E, Raju P, Rodney G. Audit of efficiency of volatile use. 2016:23-23.

14. Petre M-A. Barriers to and opportunities for recycling, reducing and reusing in Canadian academic anesthesia departments. *Canadian Journal of Anaesthesia*. 2017;(Conference: 2017 Annual meeting of the Canadian Anesthesiologists Society)

15. Webster NR. Changes are afoot. Oxford University Press; 2015. p. 1-2.

16. Morita S, Goto T, Niimi Y. Closed circuit anesthesia: a perspective in clinical practice. *Masui The Japanese Journal of Anesthesiology*. 1994;43(5):746-752.

17. Davies J, Grobler S. Coordinated eco-leadership can unite clinicians towards net zero. *BMJ*. 2021;375

18. Brooks P, Snell A, Chung C. Delivering medicines optimisation in paediatric theatres. 2019:33-33.

19. Schuster M, Richter H, Pecher S, Koch S, Coburn M. Ecological sustainability in anaesthesiology and intensive care medicine. A DGAI and BDA position aper with specific recommendations. *Anasthesiologie & Intensivmedizin*. 2020;61:329-339.

20. Huss N, Ikiugu M, Hackett F, Sheffield P, Palipane N, Groome J. Education for sustainable health care: from learning to professional practice. *Med Teach*. 2020;42(10):1097-1101.

21. Wasowicz M, Wong K, Grewal D, et al. Efficacy of simplie scavenging sysyem applied for volatile-based, long term ICU sedation. *Crit Care Med*. 2014;42(12):A1382.

22. Kennedy R, Currant P. Eighteen years of exploring patterns of anaesthetic gas flows with more recent data on CO2 GWP footprint. 2020:

23. Luo OD, Carson JJK, Sanderson V, Wu K, Vincent R. Empowering health-care learners to take action towards embedding environmental sustainability into health-care systems. *Lancet Planet Health*. 2021;5(6):e325-e326.

24. Zhong G, Abbas A, Jones J, Kong S, McCulloch T. Environmental and economic impact of using increased fresh gas flow to reduce carbon dioxide absorbent consumption in the absence of inhalational anaesthetics. *Br J Anaesth*. 2020;125(5):773-778.

25. Kole T. Environmental and occupational hazards of the anesthesia workplace. *AANA J*. 1990;58(5):327-331.

26. McGain F, Muret J, Lawson C, Sherman JD. Environmental sustainability in anaesthesia and critical care. *Br J Anaesth*. 2020;125(5):680-692.

27. Albertini P, Mainardi P, Montuori P, Sardelli P, Triassi M. Evaluation of the environmental monitoring's effects on the anaesthetic gases concentrations in the operating theatres. *Ann Ig*. 2008;20(5):455-63.

28. Feldman J, Philip J, Billard V, et al. Gas Man is an in silico translational tool to improve care and reduce inhalation anesthetic waste, cost, and pollution. 2021:620-621.

29. Navedo A, McClain C, Sullivan C, Holzman R. Go with the flow: a two-week low flow and closed circuit anesthesia elective for pediatric anesthesia clinical fellos: experience of the first two years. 2012:

30. Jarousse L. Greening the OR. *Hospitals and Health Networks*. 2015;

31. Lusby E, Connal S, Hodgetts A. How low can you go? A survey of current practice as the first step towards sustainable anaesthesia in a district general hospital. 2019:39-39.

32. Brooks P, Dunn J, Breeze J. Improving training in paediatric total intravenous anaesthesia using experience-based co-design methodology. 2020:13-13.

33. Starr SA, Baysinger CL. Inhaled nitrous oxide for labor analgesia. *Anesthesiol Clin*. 2013;31(3):623-634.

34. Beloeil H, Albaladejo P. Initiatives to broaden safety concerns in anaesthetic practice: the green operating room. *Best Pract Res Clin*. 2021;35(1):83-91.

35. Spiess W. Minimal flow anaesthesia. A modern alternative for daily practice. [German]. *Anaesthesiologie und Reanimation*. 1980;5(3):145-159.

36. Sorrentino E, Crossland C, Uncles D. Myth and reality: a survey to determine anaesthetists' knowledge and attitudes governing low-flow anaesthesia. 2011:44-44.

37. Schneck H. Nitrous oxide - 200 years of history, a splendid present, doubtful future? *Journal of Anaesthesiology Clinical Pharmacology* 2002;18(4):357-362.

38. Sartini M, Ottria G, Dallera M, Spagnolo A, Cristina ML. Nitrous oxide pollution in operating theatres in relation to the type of leakage and the number of efficacious air exchanges per hour. *J Prev Med Hyg*. 2006;47(4):155-159.

39. Nnaji C. Nitrous oxide: occupational hazards. *JBI Evidence Summary*. 2021;

40. Davies J, Grobler S, Story D. Operation clean up in australian operating theatres - a quality improvement program by TRA2SH. *Anaesthesia and Intensive Care* 2021;49(2)doi:10.1177/0310057X211055028

41. Minooee S. Perioperative settings: reuse and waste reduction. *JBI Evidence Summary*. 2021;

42. Aginga C. Perioperative settings: reusing for waste reduction. *JBI Evidence Summary*. 2021;

43. Barberio JC, Boly JD, Austin PN, Craig WJ. Pollution of ambient air by volatile anesthetics: a comparison of 4 anesthetic management techniques. *AANA J*. 2006;74(2)

44. Keen R. Pollution of operating theatres, flammable agents and other matters. *Anaesthesia*. 1980;35(7):716-717.

45. White S, Shelton C, Gelb A, et al. Principles of environmentally-sustainable anaesthesia: a global consensus statement from the World Federation of Societies of Anaesthesiologists. *Anaesthesia*. 2022;

46. Signorini A, Toffoletto F, Salvoni A, et al. Proposal of a health education program [Italian]. *Giornale Italiano di Medicina del Lavoro* 1992;14(1):97-99.

47. MacKenzie L, O'Carroll J, Arnold F, Brigue U. Recognising our carbon footprint in anaesthesia and the potential for improvements. 2018:56-56.

48. Jandu A. Recycling and greener anaesthesia at Mid Essex Hospital NHS Trust: quality-improvement project. 2020:30-30.

49. Selvaraju K, Mittal R, Nalawaya P. Recycling in anaesthetic rooms: striving for a sustainable environment. 2018:108-108.

50. Whitehead N, Brooks P, Margiotta G, Lignos L. Reducing carbon emissions: one gas at a time. *Anaesthesia*. 2021:24-24.

51. Craven T, Dua K, Evans E, O'Donohoe E. Sustainability in general anaesthesia: attitudes and capability regarding inhalational gas versus total intravenous anaesthesia. 2021:19-19.

52. Prielipp RC, Cohen NH. The future of anesthesiology: implications of the changing healthcare environment. *Curr Opin Anesthesiol*. 2016;29(2):198-205.

53. Henderson K, Raj N, Hall JE. The use of nitrous oxide in anaesthetic practice: a questionnaire survey. *Anaesthesia*. 2002;57(12):1155-1158.

54. Campbell S, Shabiyulla R, Grant A. Towards greener anaesthesia: a project to reduce volatile anaesthetic use in a large teaching hospital. 2021:94-94.

55. Baures E, Lefebure A. Train healthcare professionals for environmental sustainability of health services. *Port J Public Health*. 2022;39:31.

56. Citerio G, Bombino M, Citerio P. Training in hygiene for prevention of environmental pollution by anesthetic gases [Italian]. *Acta Toxicologica et Therapeutica*. 1985;6(1):37-44.

57. Benhamou D, Constant I, Longrois D, Molliex S, Nouette-Gaulain K, Bull A. Use of volatile anaesthetic agents in anaesthesia: a survey of practice in France in 2012. *Anaesth Crit Care Pain Med*. 2015;34(4):205-209.
